# Editorial: Decoding complexity: new insights into the cellular and molecular mechanisms of cardiovascular and metabolic diseases

**DOI:** 10.3389/fcvm.2023.1239170

**Published:** 2023-07-10

**Authors:** Chia-Feng Liu, Kailash Gulshan, Jayati Basu, Yu-Yo Sun

**Affiliations:** ^1^ Department of Cardiovascular & Metabolic Sciences, Cleveland Clinic, Cleveland, OH, United States; ^2^ Center for Gene Regulation in Health and Disease, Cleveland State University, Cleveland, OH, United States; ^3^ Department of Inflammation & Immunity, Cleveland Clinic, Cleveland, OH, United States; ^4^ Institute of Biopharmaceutical Sciences, National Sun Yat-Sen University, Kaohsiung, Taiwan

**Keywords:** aging, CVD, epigenetics, atherosclerosis, nucleolus stress, comorbidity, cardiomyopahty, heart failure

**Editorial on the Research Topic** Decoding complexity: new insights into the cellular and molecular mechanisms of cardiovascular and metabolic diseases

The collection of research articles presented here provides a comprehensive exploration of the cellular and molecular mechanisms underlying cardiovascular and metabolic diseases. The research underscores the complexity of these chronic diseases and the need for a multi-faceted approach to understanding and addressing these conditions. The articles explore the roles of periodontitis, obesity, hypercholesterolemia, and nucleolar stress in cardiovascular diseases (CVDs), offering novel insights into the intricate interplay of several factors contributing to these conditions.

Omar et al. present a novel mechanism linking periodontitis, a common oral disease, to the atherosclerotic CVD (Omar et al). The authors propose that periodontitis induces epigenetic changes in hematopoietic stem cells in the bone marrow, which persist even after the clinical elimination of the disease, thereby increasing CVD risk. This study underscores the importance of oral health in overall cardiovascular health and suggests that more research is needed to fully understand the long-term association between periodontitis and atherosclerotic CVD. Epigenetic modifications represent a promising target for the treatment of various human diseases, including CVDs (1). These findings further underscore the potential of targeting epigenetic modifications in the prevention and management of CVDs.

Aging elevates cardiovascular disease risk due to arterial stiffening, heart muscle changes, and cumulative exposure to lifestyle-related risk factors (2, 3). While aging of the cardiovascular system is an inevitable process, factors such as lifestyle choices—notably diet and physical activity levels—play a significant role in affecting this complex progression. Semeraro et al. delve into the effects of a hypercaloric high-fat diet and regular exercise on cardiac cells and aging (Semeraro et al). The study finds that while long-term moderate endurance exercise can partially reverse the effects of a high-fat diet, it may also trigger cardiac remodeling in the context of obesity. This research emphasizes the complex interplay between diet, exercise, and cardiac health, and suggests that more nuanced approaches may be needed to address obesity-related cardiac aging. The findings also highlight the need for personalized exercise prescriptions and dietary recommendations to optimize cardiovascular health in individuals with obesity.

Atherosclerosis, a leading cause of cardiovascular disease, results in the constriction of crucial arteries, consequently causing conditions such as coronary heart disease, stroke, and peripheral arterial diseases. Delving deeper into the mechanisms that underpin atherosclerosis could offer significant insights, potentially leading to the development of more effective treatments. In this context, Ghai et al. focus on the role of Heat shock factor 1 (HSF1) as a prominent cardiovascular risk factor (Ghai et al). The authors discuss how HSF1, a critical transcriptional factor of the proteotoxic stress response, is involved in lipid metabolism and cholesterol synthesis, both of which are key processes in the initiation of atherosclerosis. This review emphasizes the need for further research into the roles of HSF1 and heat shock proteins in the metabolic pathways of atherosclerosis. The insights provided in this review could pave the way for the development of novel therapeutic strategies targeting HSF1 and heat shock proteins to prevent or slowdown the progression of atherosclerosis.

The nucleolus, a pivotal cellular structure, oversees ribosome biogenesis, is crucial for protein synthesis, and regulates various other cellular processes including stress response (4). The review article by Yan and Hua explores the emerging role of nucleolar stress in the progression of cardiac diseases (Yan and Hua). The authors discuss how disruptions in ribosome biogenesis in the nucleolus can lead to nucleolar stress and initiate stress-responsive pathways that play a crucial role in the pathogenesis of cardiomyopathy. This review highlights the importance of understanding the role of nucleolar abnormalities in the development of cardiac diseases. The findings suggest that targeting nucleolar stress could be a promising strategy for the prevention and treatment of cardiomyopathy.

Collectively, these articles underscore the multifactorial nature of cardiovascular and metabolic diseases and the need for a comprehensive approach to study, prevent, and treat them. They highlight the importance of considering factors beyond traditional risk factors, such as oral health and nucleolar stress, in our understanding of these diseases. They also emphasize the complexity of the relationships between diet, exercise, and cardiac health. As we continue to unravel these complexities, we will be better equipped to develop more effective strategies for preventing and treating cardiovascular and metabolic diseases. The research presented in this collection not only advances our understanding of the cellular and molecular mechanisms underlying these diseases but also provides a solid foundation for future research in this field.
